# Differential Predictive Capabilities of Cognitive Performance on Psychological Pain Variables in Chronic Low Back Pain

**DOI:** 10.1155/prm/3164609

**Published:** 2026-09-10

**Authors:** Cory Alcon, Cassidy Krieger, Christina Piluso

**Affiliations:** ^1^ Department of Physical Therapy, High Point University, High Point, North Carolina, USA, highpoint.edu; ^2^ Department of Neuroscience, High Point University, High Point, North Carolina, USA, highpoint.edu

**Keywords:** chronic pain, cognitive function, kinesiophobia, pain catastrophizing, pain management

## Abstract

**Background:**

Chronic low back pain (CLBP) is frequently accompanied by both maladaptive pain‐related behaviors and alterations in cognitive processing. Although these domains are often studied independently, their interdependence, particularly whether cognitive performance may contribute to variability in pain‐related psychological constructs, remains insufficiently characterized.

**Methods:**

In this cross‐sectional investigation, 60 adults with CLBP completed validated questionnaires assessing pain catastrophizing (PCS), fear of movement (TSK), and symptoms associated with central sensitization (CSI). Cognitive function was evaluated across multiple domains, including attentional interference (Stroop Color Word Test), cognitive flexibility (CTMT2), and processing speed (Coding). Correlation and hierarchical regression analyses were performed to examine relationships and predictive effects while accounting for demographic and clinical factors.

**Results:**

PCS demonstrated consistent associations with reduced performance across all cognitive domains. Fear of movement was selectively related to attentional interference and processing speed, whereas central sensitization showed limited direct correlations. In regression models, cognitive flexibility (set‐shifting) and processing speed significantly contributed to the prediction of catastrophizing, while only set‐shifting independently predicted central sensitization.

**Discussion:**

These findings indicate that specific aspects of executive function—particularly cognitive flexibility—may play a meaningful role in shaping maladaptive pain‐related cognitions. Incorporating cognitive assessment into clinical evaluation may provide additional insight into individual differences in pain presentation and treatment responsiveness.

## 1. Introduction

Chronic musculoskeletal pain is increasingly conceptualized as a multifaceted condition arising from interactions among biological, psychological, and cognitive mechanisms [1–4]. Within this framework, maladaptive pain‐related responses such as pain catastrophizing (PCS), fear of movement, and central sensitization are recognized as key contributors to prolonged symptoms and functional limitation [5, 6]. PCS reflects a pattern of negatively biased thinking characterized by persistent attention to pain, exaggerated perceptions of threat, and diminished perceived control [5, 7–9]. Fear of movement, or kinesiophobia, manifests as avoidance behaviors driven by concern for injury or symptom exacerbation. Central sensitization, by contrast, involves alterations in central nervous system processing that amplify pain perception beyond what would be expected based on peripheral input alone [10, 11]. Although these constructs are often examined separately, they frequently coexist and may reinforce one another over time.

Persistent pain is also associated with measurable alterations in cognitive performance. One proposed explanation is that ongoing pain processing competes for limited cognitive resources, thereby disrupting higher‐order functions such as attentional control, working memory, and cognitive flexibility [12]. Deficits in performing tasks that require attentional shifting and the ability to selectively inhibit extraneous stimuli indicate that pain may be a predominant focus of attentional resources, leaving limited reserve for other cognitively demanding tasks [13]. When comparing chronic pain patients with age‐matched pain‐free controls, those with chronic pain performed significantly worse than controls on measures of selective attention, processing speed, executive function, and long‐term memory [14]. Similar deficits in attentional control also are found in patients with high PCS [15]. Furthermore, impaired cognitive flexibility, attentional inhibition, attentional interference, learning difficulty, and memory deficits have been associated with high levels of PCS [16, 17]. Importantly, disruptions in cognitive performance may not simply be a consequence of chronic pain but could also contribute to the persistence of maladaptive pain responses. For example, reduced cognitive flexibility may interfere with the ability to reinterpret pain experiences or disengage from threatening stimuli, while diminished processing efficiency may impair adaptive decision‐making.

Recent evidence has shown that cognitive performance is significantly poorer in those with high levels of PCS compared to low, whereas only attentional inference differed between high and low fear groups. No differences in performance were found between high and low central sensitization groups [18]. These findings substantiate the idea that pain behaviors, primarily PCS, and cognitive function heavily influence one another in those with chronic low back pain (CLBP).

Despite growing recognition of these relationships, most prior research has focused on how pain‐related behaviors influence cognition. Less attention has been given to the possibility that baseline cognitive capacity may shape the development or severity of these behaviors. Understanding this directionality may provide insight into why some individuals develop persistent pain‐related distress while others do not. Accordingly, the purpose of this study was to determine whether objective measures of cognitive performance predict levels of PCS, fear of movement, and central sensitization in individuals with CLBP.

## 2. Methods

This study used a cross‐sectional design to examine relationships between cognitive performance and pain‐related psychological variables in individuals with CLBP. Adults aged 18–65 years who reported symptoms persisting longer than 3 months were eligible for participation [19]. An a priori power analysis conducted using G∗Power indicated that a minimum of 56 participants was required to detect a moderate effect size (*d* = 0.4) with statistical power set at 0.95 and an alpha level of 0.05 [15, 20, 21]. Participants were recruited from outpatient clinics, local businesses, and university‐based advertisements within the North Carolina triad region. The selected age range was intended to minimize the influence of age‐related cognitive decline on study outcomes [22, 23]. Exclusion criteria included a history of lumbar spine surgery, systemic musculoskeletal or inflammatory disease, suspected serious pathology (e.g., fracture, malignancy, and neurological disorder), or inability to tolerate the testing position for the duration of assessment. All participants provided written informed consent prior to enrollment, and the study protocol received approval from the institutional review board.

Participants attended a single in‐person session lasting approximately 1 hour. During this session, demographic and clinical information was collected, including age, sex, highest level of education, duration of symptoms, and average pain intensity over the previous 72 h.

1. Pain intensity was assessed using the Numeric Pain Rating Scale (NPRS), an 11‐point scale ranging from 0 (“*no pain*”) to 10 (“*worst imaginable pain*”). Participants reported their current, minimum, and maximum pain levels over the preceding 3 days, and an average score was calculated for analysis [24].
2. Educational attainment was recorded using a scaled classification reflecting the highest completed level of formal education. This was captured on a 5‐point Likert scale from 0 = did not graduate high school, 1 = high school graduate/equivalent, 2 = completed some college, 3 = associates or trade school degree, 4 = Bachelor’s degree, to 5 = post‐Bachelor’s degree.

### 2.1. Pain Behaviors

Three validated self‐report instruments were used to quantify pain‐related psychological constructs:

1. The PCS is a 13‐item measure assessing exaggerated negative cognitions related to pain, including rumination, magnification, and perceived helplessness. Higher scores indicate greater levels of catastrophizing [25–27].
2. This 17‐item questionnaire evaluates fear of movement and reinjury. Total scores reflect the degree to which individuals avoid movement due to pain‐related concerns [28, 29].
3. The CSI assesses symptoms consistent with central sensitization across 25 items, with higher scores representing greater symptom burden associated with central pain processing [30].

Following, each participant completed a cognitive test battery.

### 2.2. Cognitive Measures

Participants completed a battery of cognitive assessments designed to evaluate multiple domains of executive function:

1. Attentional interference was assessed using the Stroop Color Word Test (SCWT). This task requires participants to suppress an automatic reading response in order to correctly identify incongruent stimulus features, providing an index of attentional control and interference processing [31]. This study utilized a computerized version of the SCWT via the software program DirectRT (Empirisoft Corp., New York, NY). Interference was calculated by subtracting the average time needed to complete the first two tasks from the time needed to complete the third task: interference score = SCWT 3 ‐ ([SCWT 1 + SCWT 2]/2) [32–34].
2. Cognitive flexibility was evaluated using the Comprehensive Trail Making Test Second Edition (CTMT2), which requires participants to alternate between competing cognitive sets. Performance on later trials reflects the ability to efficiently shift between mental frameworks, an essential component of executive functioning [35, 36].
3. Processing speed and sustained attention were measured using the Coding subtest of the Wechsler Adult Intelligence Scale–Fourth Edition (WAIS‐IV), which requires rapid symbol–number matching under time constraints. Higher scores indicate more efficient information processing and attentional capacity [37, 38].

### 2.3. Statistical Analysis

Descriptive statistics were calculated for participants’ demographics, low back pain history, and all outcome measures. Pearson’s correlations were performed to assess the relationships between pain‐related variables (PCS, TSK, and CSI) and each cognitive performance measure. Hierarchical multiple linear regression analyses were then conducted to examine the extent to which cognitive performance predicted PCS, TSK, and CSI scores after accounting for relevant demographic and clinical covariates. For each regression model, age, sex, education level, and pain duration were entered in Step 1, followed by SCWT interference, CTMT2 inhibitory control, CTMT2 set‐shifting, and coding performance in Step 2. Model fit, standardized regression coefficients, and change in explained variance (ΔR^2^) were examined for each outcome. Collinearity diagnostics were also reviewed to assess multicollinearity. The *α* level was set at 0.05 for all analyses.

## 3. Results

Sixty participants were enrolled in the study. All participants completed all demographic, pain related, and cognitive performance measures. Table 1 displays descriptive demographic data for the sample as whole.

**TABLE 1:** Demographic characteristics of all participants.

Outcome	Mean ± SD (*n* = 60)
Age (years)	41.25 ± 15.49
Sex (male/female)	10/50
Duration (months)	135.11 ± 144.54
Education (0–5) median (IQR)	4 (2–4)
NPRS (0–10)	4.29 ± 2.03
PCS (0–52)	24.71 ± 13.89
TSK (17–68)	37.69 ± 10.53
CSI (0–100)	40.35 ± 11.97

*Note:* IQR, interquartile range.

Abbreviations: CSI, Central Sensitization Inventory; NPRS, Numeric Pain Rating Scale; PCS, Pain Catastrophizing Scale; TSK, Tampa Scale of Kinesiophobia.

Correlations between pain‐related variables and cognitive test performance are summarized in Table 2. Performance on the SCWT showed positive associations with both PCS (*r*(58) = 0.321, *p* = 0.02) and kinesiophobia (TSK; *r*(58) = 0.334, *p* = 0.013). In contrast, CTMT2 measures of inhibitory control and set‐shifting, along with coding task performance, demonstrated negative relationships with PCS (*r*(58) = −0.250, *p* = 0.047; *r*(58) = −0.498*,* *p* < 0.001; and *r*(58) = −0.525, *p* < 0.001, respectively). Additionally, coding performance was inversely correlated with TSK scores (*r*(58) = −0.281, *p* = 0.041).

**TABLE 2:** Pearson correlation coefficients examining associations between pain‐related variables and cognitive performance outcomes.

	SCWT	CTMT2‐inhibitory control	CTMT2‐set shifting	Coding
PCS	0.321^∗^	−0.250^∗^	−0.498^∗∗^	−0.525^∗∗^
TSK	0.334^∗^	−0.238	−0.176	−0.281^∗^
CSI	−0.034	0.186	−0.140	0.070

*Note:* CTMT2 – Comprehensive Trail Making Test – Second Edition. *N* = 60.

Abbreviations: CSI, Central Sensitization Inventory; PCS, Pain Catastrophizing Scale; TSK, Tampa Scale of Kinesiophobia.

^∗^ *p* < 0.05.

^∗∗^ *p* < 0.001.

Hierarchical multiple regression analyses were conducted to determine whether cognitive performance measures predicted PCS, TSK, and CSI scores after controlling for age, sex, education, and pain duration. Collinearity diagnostics indicated no evidence of problematic multicollinearity across models (VIF range = 1.02–2.60). Table 3 presents the results of the hierarchical regression analyses.

**TABLE 3:** Hierarchical multiple regression models predicting PCS, TSK, and CSI scores.

Predictor/model statistic	PCS (*β*, *p*)	TSK (*β*, *p*)	CSI (*β*, *p*)
*Step 1: covariates*			
Age	0.173, 0.185	0.219, 0.153	−0.130, 0.369
Sex/gender	−0.080, 0.475	0.081, 0.541	−0.086, 0.496
Education	−0.053, 0.657	−0.157, 0.268	−0.054, 0.689
Pain duration	−0.327, 0.014^∗^	−0.180, 0.235	0.252, 0.085

*Step 2: cognitive predictors*			
Stroop interference	0.114, 0.416	0.276, 0.095	−0.093, 0.549
CTMT2 inhibitory control	0.310, 0.056	−0.066, 0.725	0.210, 0.242
CTMT2 set‐shifting	−0.294, 0.036^∗^	0.145, 0.465	−0.691, < 0.001^∗∗^
Coding	−0.367, 0.039^∗^	−0.109, 0.594	0.366, 0.064

*Final model summary*			
*R* ^2^	0.412	0.194	0.268
Adjusted *R* ^2^	0.320	0.067	0.154
*F* for final model	4.472	1.534	2.339
*p* for final model	< 0.001^∗∗^	0.169	0.032^∗^
ΔR^2^ from Step 2	0.224	0.083	0.202
*F* change for Step 2	4.857	1.307	3.519
*p* for *F* change	0.002^∗∗^	0.280	0.013^∗^

*Note:* Standardized beta coefficients (*β*) are reported for the final model (Step 2), which included age, sex/gender, education, pain duration, Stroop interference, CTMT2 inhibitory control, CTMT2 set‐shifting, and coding. *N* = 60.

Abbreviations: CSI, Central Sensitization Inventory; PCS, Pain Catastrophizing Scale; TSK, Tampa Scale of Kinesiophobia.

^∗^ *p* < 0.05.

^∗∗^ *p* < 0.001.

For PCS, the Step 1 model including age, sex, education, and pain duration was statistically significant, accounting for 18.8% of the variance (*R* ^2^ = 0.188, *F*(4, 55) = 3.193, *p* = 0.020). The addition of cognitive variables in Step 2 significantly improved the model (ΔR^2^ = 0.224, *F* change(4, 51) = 4.857, *p* = 0.002), resulting in a final model that explained 41.2% of the variance in PCS scores (*R* ^2^ = 0.412, adjusted *R* ^2^ = 0.320, *F*(8, 51) = 4.472, *p* < 0.001). In the final model, set‐shifting and coding performance were significant independent predictors of PCS scores (*β* = −0.294, *p* = 0.036, and *β* = −0.367, *p* = 0.039 respectively).

For TSK, the Step 1 model was not significant (*R* ^2^ = 0.111, *F*(4, 55) = 1.722, *p* = 0.158), and the addition of cognitive variables did not significantly improve model fit (ΔR^2^ = 0.083, *F* change(4, 51) = 1.307, *p* = 0.280). The final model was not statistically significant (*R* ^2^ = 0.194, adjusted *R* ^2^ = 0.067, *F*(8, 51) = 1.534, *p* = 0.169), and no individual predictors reached significance.

For CSI, the Step 1 model was not significant (*R* ^2^ = 0.067, *F*(4, 55) = 0.981, *p* = 0.425). However, the addition of cognitive variables in Step 2 significantly improved the model (ΔR^2^ = 0.202, *F* change (4, 51) = 3.519, *p* = 0.013). The final model was significant and explained 26.8% of the variance in CSI scores (*R* ^2^ = 0.268, adjusted *R* ^2^ = 0.154, *F*(8, 51) = 2.339, *p* = 0.032). In the final model, CTMT2 set‐shifting was the only significant predictor of CSI scores (*β* = −0.691, *p* < 0.001). The results of the hierarchical regression analyses examining the predictive capabilities of cognitive performance variables are summarized in Table 3.

## 4. Discussion

The purpose of this study was to explore whether cognitive performance metrics can predict variability in pain‐related psychological constructs in individuals with CLBP. The findings suggest that specific domains of cognitive function, particularly set‐shifting and processing speed, are associated with higher levels of PCS and central sensitization. These findings extend prior work examining the relationship between pain behaviors and cognitive performance by suggesting that this relationship may be bidirectional, with cognitive capacity potentially influencing the development or continuation of maladaptive pain responses.

Set‐shifting is a key aspect of cognitive flexibility, which is the ability to adapt to changing demands and switch between different tasks or mental processes [39, 40]. Studies suggest that individuals with CMP may experience impairments in cognitive flexibility, particularly set‐shifting abilities [41, 42]. It appears that pain may disrupt attention and focus, making it more difficult to switch between tasks [43]. This may cause difficulty with daily function where adaptability to changing situations and multitasking is required [44]. There also appears to be a significant relationship between attentional control and PCS as those who catastrophize may have difficulty shifting their attention away from their pain [45, 46]. Our findings agree with the previous literature in that poorer set‐shifting abilities predict higher levels of PCS, further emphasizing the dynamic relationship between the two variables.

We also found that performance on the coding test was predictive of PCS levels. The coding test is commonly used to assess neurological and cognitive dysfunction, specifically attentional control, processing speed, and memory [47]. Poorer coding test performance has been associated with CMP and with levels of PCS [48–50]. Our results found coding test performance to be a more sensitive predictor of PCS levels. This may be due to the more sensitive nature of the test itself as it is thought to capture a broader spectrum of cognitive dysfunction than other tests [51, 52]. This may provide evidence for the use of this test as an initial screening measure in those with CMP appearing with cognitive difficulties.

Only the CTMT2 set‐shifting was a significant predictor of central sensitivity levels in this study. Again, set‐shifting appears to be an acceptable measure of how well someone can cognitively manage multiple thought processes or tasks. Central sensitization is commonly associated with “brain fog” or difficulty concentrating, along with impaired memory and attention [53–55]. It appears that central sensitization and cognitive performance are interconnected as dysregulation of one can affect the other. Central sensitization is an imbalance in the nervous system’s pain‐modulating system, which results in more sensitive pain perception and potentially dysfunctional cognitive processing [56]. Therefore, those with poor set‐shifting abilities may enhance an already sensitive nervous system’s ability to further processing incoming information.

Research indicates that pre‐existing cognitive impairments, such as difficulties with memory, attention, or cognitive flexibility, can be linked to the development of chronic pain [57, 58]. However, the results from this study are the first to indicate that cognitive dysfunction may also predict negative pain behaviors that likely influence the progression and severity of one’s pain complaint. Given the high frequency of cognitive impairment in those CMP, increased clinical assessment is likely warranted [59].

There are several limitations to this study. While the age range of 18–65 was selected to control for age‐related cognitive decline, findings in those younger than 18 could not be accounted for. There is evidence to suggest similar relationships between PCS and cognitive function in adolescents to those found in this study [16]. However, these could not be replicated in the current study. Second, a majority of the participants enrolled in this study were female (see Table 1). Gender differences in PCS have been found with females typically reporting higher levels than men [60, 61]. Thus, an understanding of potential differences in relationships between the study variables between males and females cannot be stated. Finally, we studied the predictive abilities of cognitive performance on pain behaviors. It is just as likely that pain behaviors may predict cognitive performance considering that the interference resulting from maladaptive thoughts and beliefs may hinder cognitive performance. Thus, future research efforts should address this side of the relationship.

The findings from this study continue to support the presence of a relationship between pain behaviors and cognitive function. It appears that cognitive performance, specifically set‐shifting abilities, processing speeds, and sustained working memory predict the presence of PCS and central sensitization in participants with CLBP. This distinction is clinically meaningful, as it shifts cognitive dysfunction from being viewed solely as a consequence of chronic pain to a potential contributing factor in its psychological and behavioral manifestations. These findings highlight the importance of cognitive dysfunction and maladaptive pain behaviors; two potential areas that interventions may impact when managing those with CLBP as well a potentially underevaluated set of symptoms in the clinical setting.

## Author Contributions

Cory Alcon contributed to study design data collection, data analysis, and manuscript preparation. Cassidy Krieger contributed to data collection, data analysis, and manuscript preparation. Christina Piluso contributed to data collection and analysis.

## Funding

There are no funding sources to report.

## Ethics Statement

Institutional Review Board (IRB) approval was received from the High Point University IRB committee on 7/13/2023 (IRB‐FY2023‐122).

## Consent

Consent was obtained from each participant in this study as the minimum age to participate was 18 years of age.

## Conflicts of Interest

The authors declare no conflicts of interest.

## Data Availability

Data from this study will be made available on request from the corresponding author.
